# The use of predictive models to develop chromatography-based purification processes

**DOI:** 10.3389/fbioe.2022.1009102

**Published:** 2022-10-12

**Authors:** C. R. Bernau, M. Knödler, J. Emonts, R. C. Jäpel, J. F. Buyel

**Affiliations:** ^1^ Fraunhofer Institute for Molecular Biology and Applied Ecology IME, Aachen, Germany; ^2^ Institute for Molecular Biotechnology, RWTH Aachen University, Aachen, Germany; ^3^ University of Natural Resources and Life Sciences, Vienna (BOKU), Department of Biotechnology (DBT), Institute of Bioprocess Science and Engineering (IBSE), Vienna, Austria

**Keywords:** biopharmaceutical production process, Data-driven models, downstream processing design, experiment quality, hybrid model validation, mechanistic modeling, protein separation, quality by design

## Abstract

Chromatography is the workhorse of biopharmaceutical downstream processing because it can selectively enrich a target product while removing impurities from complex feed streams. This is achieved by exploiting differences in molecular properties, such as size, charge and hydrophobicity (alone or in different combinations). Accordingly, many parameters must be tested during process development in order to maximize product purity and recovery, including resin and ligand types, conductivity, pH, gradient profiles, and the sequence of separation operations. The number of possible experimental conditions quickly becomes unmanageable. Although the range of suitable conditions can be narrowed based on experience, the time and cost of the work remain high even when using high-throughput laboratory automation. In contrast, chromatography modeling using inexpensive, parallelized computer hardware can provide expert knowledge, predicting conditions that achieve high purity and efficient recovery. The prediction of suitable conditions *in silico* reduces the number of empirical tests required and provides in-depth process understanding, which is recommended by regulatory authorities. In this article, we discuss the benefits and specific challenges of chromatography modeling. We describe the experimental characterization of chromatography devices and settings prior to modeling, such as the determination of column porosity. We also consider the challenges that must be overcome when models are set up and calibrated, including the cross-validation and verification of data-driven and hybrid (combined data-driven and mechanistic) models. This review will therefore support researchers intending to establish a chromatography modeling workflow in their laboratory.

## 1 Introduction

Chromatography is the method of choice for the purification of biopharmaceutical proteins during downstream processing (DSP), typically achieving a purity >95% for the final product (Geigert 2013). However, chromatography is also a major cost driver during production and process development due to the high cost of materials and the time-consuming optimization of process conditions to increase the yield, recovery and purity while minimizing the environmental impact (Buyel and Fischer 2014; Madabhushi et al., 2018). Optimization focuses on the interaction between a protein and a ligand-coated stationary phase within a chromatography column, which can be modified by adjusting factors such as the resin matrix, pore size, ligand type and density, pH, flow rate, temperature and conductivity, thus constituting a multi-parameter problem (Schmidt-Traub et al., 2020). A combination of automated, high-throughput screening and scale-down models (SDMs) can reduce the time required and therefore the cost of optimization, and can be used to build empirical, data-driven descriptive models (Kawajiri 2021), for example using a design of experiments (DoE) approach (Hibbert 2012a; Bayer et al., 2021). Nevertheless, the identification and quantification of key factors and their interactions is dependent on substantial infrastructure and experimental effort, and the efficiency of the process can largely depend on the availability of experienced staff (Hanke and Ottens 2014).

In contrast, mechanistic models describe chromatography based on physicochemical principles and thus facilitate *a priori* predictions about protein separation processes (Shekhawat and Rathore 2019). These models consist of equations describing mass transport (e.g., the general rate model (Püttmann et al., 2013), and protein sorption (e.g., the steric mass action (SMA) model (Brooks and Cramer 1992; Osberghaus et al., 2012a). Mechanistic models require calibration with empirical data, such as resin-specific gradient elution experiments and breakthrough curves (Schmidt-Traub 2006), and they need substantial computational power (Juliane Dorothea Diedrich 2019; Rischawy et al., 2019). Accordingly, these models are currently used mainly for late-stage downstream process characterization but their widespread use in academia and industry is limited by the complexity of model calibration and implementation (Saleh et al., 2020). Nevertheless, there is a growing commercial interest in the topic because it can accelerate process development (Mouellef et al., 2021), for example, the German start-up company GoSilico has developed chromatography modeling software (Hahn et al., 2012) and was acquired by Cytiva (formerly GE Healthcare) in 2021^1^. Specifically, mechanistic modelling can improve holistic process understanding (Close 2015), increase transferability to new processes, and simplify change management (Djuris and Djuric 2017). For example, the experimental effort required to model a cation exchange chromatography step for a monoclonal antibody *in silico* was reduced by ∼75% compared to traditional laboratory-based process characterization (Saleh et al., 2021c). This reflected the ability of the model to predict the effect of changes in protein surface charge on separation *a priori*, thus accounting for the impact on purification (Saleh et al., 2021a). Similarly, mechanistic models can augment SDM-based data by incorporating process information about loading density, bed height or mobile phase properties (Saleh et al., 2021b). Furthermore, consecutive, orthogonal purification steps can be optimized in a holistic manner, for example by ensuring compatibility between the elution conditions of the first step and the loading conditions of the next (Huuk et al., 2014).

Ultimately, mechanistic models may be combined with data-driven counterparts to form hybrid models that can build the basis of a digital twin for a production process. Such a twin can be augmented through real-time data from process analytical technologies (PAT) to facilitate model-predictive control (MPC) as a means to ensure continuous optimal performance (Mollerup et al., 2008; Andris and Hubbuch 2020; Saleh et al., 2021c; Moser et al., 2021). This is in line with the quality by design (QbD) approach to improve process robustness and consistency by ensuring fundamental process understanding (Shekhawat et al., 2016; Saleh et al., 2021c). Thereby, the different types of chromatography models support a risk-based approach during pharmaceutical product and process development as proposed by the European Medicines Agency (EMA) and US Food and Drug Administration (FDA), and as outlined in the International Council for Harmonisation (ICH) quality guidelines Q8–Q11 (Holm et al., 2017).

In this article, we consider the requirements and challenges associated with data-driven, mechanistic and hybrid modeling of chromatography, which we introduce first. Then, we discuss the individual challenges starting with interdisciplinary work necessary to build such models and to collect high-quality experimental data for model calibration. We also highlight the benefits and limitations of the different modeling approaches, including model calibration, parameter fitting and validation, and the impact of nonspecific protein–resin interactions. However, the design and optimization of multi-stage purification processes is beyond the scope of this article and is not discussed any further.

## 2 Modeling approaches

### 2.1 Descriptive models

Data-driven models can be built without prior knowledge of the mechanisms underlying the process under investigation and are therefore especially useful for poorly characterized settings, for example in the social sciences (Stattner and Collard 2015a) or in complex biotechnological production processes (Walther et al., 2022a). A pre-defined set of equations is not required to account explicitly for all the proteins in a chromatographic separation. Instead, data-driven approaches use (experimental) data to build descriptive or predictive models *a posteriori* by applying data analysis techniques such as machine learning (ML) and classical statistical regression analysis (Mitchel 1997a; Vidakovic 2011; Song et al., 2021b). The latter can be applied retrospectively, for example by subjecting the data to principal component analysis (PCA), which can also be considered as a form of ML, or principal component regression (PCR) (Jolliffe and Cadima 2016a). These operations reduce the dimensionality of the data and establish correlations between a dependent variable (e.g., an isotherm parameter) and independent variables (e.g., chromatography conditions) that were measured during the experiments. Alternatively, explorative data analysis can be used to identify potential correlations within the data *ex post*, for example visual inspection by a data scientist and process engineer or mathematical approaches such as independent principal component analysis (IPCA) (Yao et al., 2012a). The results can guide the selection of suitable models for subsequent parameter fitting. For example, linear or non-linear functions (Sahinidis 2019a) may be identified that describe the shape of chromatogram peaks, as shown using an exponentially-modified Gauss (EMG) function (Kalambet et al., 2011a), and the multiscale optimization of an antibody purification process (Liu and Papageorgiou 2019b). Data-driven models can also be designed in a structured manner by defining the corresponding experiments *ex ante*. A prominent example is the design of experiment (DoE) approach (Mandenius and Brundin 2008a; Hibbert 2012a; Ganorkar and Shirkhedkar 2017a; Möller et al., 2019a), in which data points are optimally positioned in a multi-dimensional space constrained by the independent variables to facilitate the fitting of a multiple linear regression (MLR) model of pre-defined maximal complexity (e.g., a cubic model). Once the experimental data have been collected, an analysis of variance (ANOVA) is conducted to remove non-significant terms from the model, unless required to maintain hierarchy (Peixoto 1987b). The resulting models can be used for the simple optimization of chromatography conditions and other separation operations based on the specific product, feed or resin, with little experimental and analytical effort. However, DoE (or more precisely, the underlying ANOVA and quality control tools) typically does not work well with heterogeneous data (e.g., non-normally distributed or non-homoscedastic data) and/or large datasets (>200 data points) that contain multiple, local optima, resulting in a complex surface (Osberghaus et al., 2012c).

For complex datasets, unsupervised, supervised and reinforced ML methods tend to perform better than classical statistics (Bishop 2016). Reinforced ML methods are currently of little interest in chromatography modeling because they require a feedback loop between the ML model and a physical experimentation unit generating new data, although this may become possible in the near future. Unsupervised learning can use, for example, PCA or a support vector machine (SVM) to reveal the internal structure of a dataset, thus facilitating a reduction in dimensionality. Supervised learning is currently the most suitable ML approach for chromatography modeling. Here, an initial dataset of independent and dependent variables, for example in the context of quantitative structure–activity relationship (QSAR) modeling (Tropsha and Golbraikh 2007b), is divided into a training set and a test set. The training dataset is used to train a mathematical model, and the model predictions can then be compared with the test dataset to assess the quality of the trained model, for example to detect overfitting (Bishop 2016). The model can be build using various approaches, including clustering methods such as artificial neural networks (ANNs), random forests or decision trees, but may also make use of regression for some operations, such as partial least squares regression, support vector regression (SVR), least absolute shrinkage and selection operator (LASSO) regression or ridge regression (Tibshirani 1996b; Dasgupta et al., 2011a). In this context, a major difference between statistical analysis and ML is that the latter often sacrifices interpretability (or explainability) in favor of the model’s predictive power (Song et al., 2021b), even though both ML and statistical analysis may perform equally well on some datasets. For example, area under the receiver operation characteristic (AUROC) curves have been compared for models predicting various diseases, revealing values of 0.736 (ML) vs*.* 0.748 (logistic regression) when predicting acute kidney disease (Song et al., 2021b), 0.837 (neural net models) vs*.* 0.836 (regression models) when predicting infraction mortality (Piros et al., 2019a), and 0.926 (ANN) vs*.* 0.869 (Cox regression) when predicting the outcome of COVID-19 (Abdulaal et al., 2020b).

Data-driven models have been widely used in the context of chromatography. In one case, the separation of herbal extract compounds was modeled by first using a DoE approach to generate experimental data and then correlating the responses with the separation conditions using a regular MLR model but also a SVM and ANN, all of which yielded similar predictions with a Pearson’s correlation coefficient *r* > 0.99 (Ge et al., 2021b). In another study, SVR was used to link the properties of synthetic nucleotides (e.g., hairpin structures) with their retention times on a phenyl column (Enmark et al., 2022a). The root-mean-square error (RMSE) between observed and predicted peak maxima was used as a model quality indicator for different types of gradients, and the corresponding determination coefficients for the training (R^2^) and test (Q^2^) datasets were >0.99, whereas an empirical logarithmic model achieved values as low as 0.93 (R^2^) and 0.85 (Q^2^) depending on the chromatography setting. Similarly, ML has been used to model the purification of inclusion bodies (Walther et al., 2022a) and antibodies (Robinson et al., 2017b), to predict antibody retention on a hydrophobic interaction chromatography (HIC) resin (Jain et al., 2017b), to improve peak detection (Chetnik et al., 2020a), and to predict the elution behavior of host cell proteins (HCPs) from an ion-exchange matrix (Buyel et al., 2013) as well as to fit SMA isotherm parameters (Jäpel and Buyel 2022).

The validation of data-driven models is important to prevent overfitting. A detailed analysis of quality assessment procedures for data-driven models is beyond the scope of this review, but typical elements include bootstrapping (resampling with replacement), *k*-fold cross-validation (leave-*x*-out, resampling without replacement) (Kim 2009b), *y*-randomization (*y*-scrambling, random assignment of the dependent variables to the dependent ones) and the use of an additional external dataset (independent of the test dataset) (Tropsha et al., 2003). The prediction and extrapolation or applicability domains of the model should also be defined to prevent inappropriate use (Tropsha et al., 2003; Gramatica 2007). It is important to note that data-driven models are typically only valid within the parameter space (or a fraction thereof) constrained by the elements in the training dataset, so they do not allow extrapolation (Tropsha and Golbraikh 2007b). Specifically, data-driven models cannot make *de novo* predictions because they cannot go beyond the content of the underlying dataset (Tropsha and Golbraikh 2007b), as is the case for protein structure prediction using AlphaFold (Jumper et al., 2021). Accordingly, the test dataset should be within the applicability domain of the training dataset. Despite these quality assurance measures, it can be difficult to reproduce specific ML results because there are many different meta-parameters to select, which is why reproducibility standards are often necessary to ensure reliability (Heil et al., 2021b).

Data-driven models could be improved in several ways in the future. For example, PCA is currently limited to datasets with 1000–2000 entries, which is considered ‘large’ (Vogt and Tacke 2001a; Rachakonda et al., 2016a), but is probably small compared to the anticipated results of high-throughput experiments and the content of community-based databases. Therefore, efficient ways will be required to handle ‘big data’, such as healthcare patient data (Ahmed et al., 2021a; Dong et al., 2021b).

### 2.2 Mechanistic models

In contrast to purely data-driven descriptive models, mechanistic models aim to simulate actual physicochemical mechanisms based on mathematical equations and, once calibrated with a defined set of bind-and-elute gradients and breakthrough curves (Section 4.2), such models allow the extrapolation of separation processes *in silico* for conditions outside the parameter space tested experimentally (Benner et al., 2019; Kumar and Lenhoff 2020).

In order to set up a mechanistic chromatography model for a specific set of conditions, e.g., resin and ligand type, column dimensions and operation parameters as well as proteins to be separated, equations for mass transfer and sorption must be defined. Mass transfer has been studied in detail and can be described precisely using different formulae, such as the transport dispersive model (TDM), equilibrium dispersive model (EDM) or the general rate model (GRM), the latter probably being the most prominent and widely used (Schmidt-Traub 2006; Shekhawat and Rathore 2019). Other mechanistic models describing the mass transport include the transport model, reactive-dispersive model and modified versions of the GRM such as the Thomas model (also referred as kinetic model) (Table 1). For example, the Thomas model describes the convective transport and adsorption rate kinetics while neglecting axial dispersion and mass transfer kinetics such as external and internal diffusion (Cavazzini et al., 2002; Shekhawat and Rathore 2019). The GRM captures mass transport processes both outside resin pores (Eq. 1, inter-particle mass balance) and within them (Eq. 2, intra-particle mass balance) during packed-bed chromatography.

$$\frac{\partial c_{i}}{\partial t}=-u\frac{\partial c_{i}}{\partial z}+D_{ax}\frac{\partial^{2}c_{i}}{\partial z^{2}}-\frac{1-\varepsilon_{i}}{\varepsilon_{i}}\;\frac{3}{r_{p}}k_{f,i}\left(c_{i}-c_{p,i}\left(∙,∙,r_{p}\;\right)\right)$$ (1)

where *c*_*i*_ is the concentration of colloid *i*, *u* is the linear inter-particle fluid velocity in the axial orientation *z*, *D*_*ax*_ is the axial dispersion coefficient, *ε*_*i*_ is the inter-particle porosity, *r*_*p*_ is the resin particle radius, factor 3/*r*_*p*_ accounts for its surface-to-volume ratio, *k*_*f,i*_ is the effective linear flux (i.e., volumetric flow rate divided by column cross-sectional area and inter-particle porosity) through the stagnant film zone around the stationary phase beads, and *c*_*p,i*_ is the mobile phase concentration of particle *i* ^2^.

$$\frac{\partial c_{p,i}}{\partial t}=D_{p,i}\left(\frac{\partial^{2}c_{p,i}}{\partial r^{2}}+\frac{2}{r}\frac{\partial c_{p,i}}{\partial r}\right)-\frac{1-\varepsilon_{p}}{\varepsilon_{p}}\frac{\partial q_{i}}{\partial t}$$ (2)

where ∂c_p,i_/∂t is the concentration change of colloid i in the porous bead over time, D_p,i_ is the pore diffusion coefficient of component i, with resin particle radius r, ε_p_ is the intra-particle porosity, and ∂q_i_/∂t is the change in surface-bound component i over time. Whereas the inter-particle mass balance accounts for convection, dispersion and film mass transfer, the intra-particle mass balance represents pore diffusion processes inside the mostly spherical particles of the stationary phase and the sorption of colloid *i* to the surface, which is defined by the corresponding isotherms. The calculation becomes more complex if the two-dimensional general rate model (GRM2D) is used, which includes a radial coordinate to consider non-axial transport resulting from inhomogeneous resin packing or dispersion at the frits of the column inlet (Brhane et al., 2019). This mass transport model is already implemented in some modeling software^3^. The complexity can be reduced by using a modified lumped rate model that excludes intra-particle protein binding^4^.

**TABLE 1:** List of mass transport models and adsorption kinetic models for the mechanistic modeling of the protein transport and adsorption during chromatography-based purification processes.

Mass transport models	Reference
General rate model	Lieres and Andersson (2010)
Two-dimensional general rate model	Brhane et al. (2019)
Lumped rate model	Leweke and Lieres (2018)
Transport dispersive model	Piątkowski et al. (2003)
Equilibrium dispersive model	Guiochon et al. (2006)
Thomas model (kinetic model)	Cavazzini et al. (2002)
Reactive-dispersive model	Golshan-Shirazi and Guiochon (1991)
Transport model	Qamar et al. (2020)
Adsorption kinetic models	Reference
Steric mass action model	Brooks and Cramer (1992)
Langmuir model	Guiochon (2002)
Extended Langmuir model	Kumar et al. (2015)
Exponentially modified Langmuir model	Antia and Horváth (1989)
Stoichiometric displacement model	Geng and Zebolsky (2002)
Non-ideal surface solution model	Raje and Pinto (1998)
Preferential interaction model	Perkins et al. (1997)

In addition to general isotherms such as the Freundlich formalism and Langmuir adsorption model, specific isotherms have been developed to account for different types of chromatography. Examples are the stoichiometric displacement model, the non-ideal surface solution model and preferential interaction model (Table 1). Often, modified versions of kinetic equations are used to reduce or increase the complexity of the mechanistic model e.g., as shown for the extended Langmuir model or exponentially modified Langmuir model (Table 1). The SMA model is often used to describe sorption during ion exchange chromatography (IEX) and specifically accounts for the salt concentration, number of interacting ligands, and steric shielding of the ligand by bound proteins (Parente and Wetlaufer 1986; Degerman et al., 2007; Osberghaus et al., 2012a; Bernau et al., 2021). Currently, more than 15 isotherms are available for the description of chromatography modalities such as IEX and HIC (Guo et al., 2020; Kumar and Lenhoff 2020; Saleh et al., 2021a; Saleh et al., 2021b; Kumar et al., 2021) as has been reviewed elsewhere (Wang et al., 2016; Shekhawat and Rathore 2019). In contrast, few isotherms are available for mixed-mode or multi-modal chromatography (MMC), probably due to the yet incomplete understanding of the mechanistic basis of this process (Kumar and Lenhoff 2020) and/or because the corresponding resins are often used in flow-through mode to bind HCPs.

The implementation of mathematical models requires software solutions in order to set up and calibrate the model before *in silico* prediction (Schmölder and Kaspereit 2020). The Chromatography Analysis and Design Toolkit (CADET) is a fast and accurate solver and chromatogram simulator that covers a wide range of models, including GRM variants such as GRM2D and reduced variants of the lumped rate model (Leweke and Lieres 2018; Leweke et al., 2020; Narayanan et al., 2021b). Commercially available counterparts include Cytiva’s GoSilico Chromatography Modeling Software (Briskot et al., 2021) and ChromWorks from YPSOFacto (Kaspereit and Schmidt-Traub 2020; Schmölder and Kaspereit 2020)^5^.

As for data driven models, performance can be assessed by comparing experimental data and simulation results, for example based on the sum of the squared errors (SSE) between the training and validation datasets. Other verification methods like the R^2^ and the RMSE have been described (Rajamanickam et al., 2021). Model predictions can be improved by augmenting chromatography models with parameters that account for specific physiochemical effects. For example, the SMA isotherm can be expanded to account explicitly for the impact of pH, and protein-specific pore accessibilities may be included in the GRM (Bowes et al., 2009; Coquebert de Neuville et al., 2013; Saleh et al., 2020; Frank et al., 2022)^6^. However, with an increasing number of model parameters even mechanistic models can be overfitted as experimental noise may unduly affect parameter calibration (Rajamanickam et al., 2021). Such overfitting often results in extreme model predictions and poor generalization (Steyerberg 2019). Furthermore, isotherm parameters may become difficult to identify unambiguously based on the experimental data, as discussed in the next section.

### 2.3 Hybrid models

It may be possible to combine ML with the knowledge-based components of mechanistic models to form hybrid models, as discussed for the multi-scale modeling of biological systems (Alber et al., 2019) and applied to the informed selection of DoE parameter ranges (Joshi et al., 2017; Möller et al., 2019a). A comparison of data-driven and mechanistic models in the context of chromatography has revealed that the former are fast and accurate within their design space with little experimental effort and simple analysis, but cannot extrapolate beyond these boundaries, whereas mechanistic models can extrapolate and achieve accurate predictions well beyond the characterized parameter space, but require much more effort to calibrate and solve (Osberghaus et al., 2012c).

Hybrid models combine data-driven, descriptive models with process knowledge captured in mechanistic models (Stosch et al., 2014). They provide better process understanding and allow extrapolation, with less demand for data quality and quantity compared to purely data-driven models (Glassey and Stosch 2018). Hybrid models also enable the use of mechanistic knowledge if the prerequisites for purely mechanistic models are not met, meaning that the mechanisms are insufficiently established in equations (Solle et al., 2017). Hybrid models described in the literature are predominantly used for upstream production (Stosch et al., 2016; Simutis and Lübbert 2017), with only a few examples of hybrid modeling in DSP (Narayanan et al., 2021b; Narayanan et al., 2021a): Namely, Narayanan et al. learned the chromatographic unit behavior by a combination of neural network and mechanistic model while fitting suitable experimental breakthrough curves (Narayanan et al., 2021b), Joshi et al. (Joshi et al., 2017) used a mechanistic model to simulate the analytical separation for the DOE and build with the results an empirical model, and Creasy et al. (Creasy et al., 2019) learned the adsorption isotherm model from batch isotherm data by using interpolation techniques.

During the development of chromatography-based purification processes, hybrid models can be used to accelerate the tedious experimental adjustment of parameters for mechanistic models (Narayanan et al., 2021a) or to reduce the quantity of data required for their calibration (Solle et al., 2017). For example, parameters of a mechanistic model such as the SMA isotherm can be predicted by a data-driven QSAR model using a small training dataset (∼30 proteins for which the SMA parameters have been determined experimentally).

## 3 Challenge I: Communication barriers can slow down interdisciplinary research

The implementation of chromatography models that account for the biochemical and physicochemical properties of a system while following the rules and operations of algebra requires a bidirectional exchange of knowledge between experimenters and data scientists. This can be hampered by differences in problem solving approaches, limited knowledge about the possibilities and limitations of the complementary scientific domain, and the use of discipline-specific terminology and jargon (Bracken and Oughton 2006; Bowman 2007; Monteiro and Keating 2009). For example, the term “transformation” has multiple distinct meanings in biology, chemistry, physics and mathematics, and it may not always be clear which sense is implied in a multidisciplinary context. There may also be differences in the interpretation of terms (e.g., a biologist may think of a certain descriptor as a scalar, whereas a data scientist may consider it also as a vector or a matrix) and in the conceptualization of tasks (e.g., experimenters typically do not think of scientific tasks as formulae, algorithms or models, whereas this is the typical expectation of mathematicians and data scientists). Finally, data scientists tend to focus on abstract, general and fundamental solutions to a (mathematical) problem, whereas process engineers focus on only those aspects of the solution that can be applied in practice. In the context of this review, interdisciplinary discussions were triggered by discrepancies affecting data quantity, quality and presentation, which can create fundamental tension in the design and analysis of experiments and subsequent model building (Pischke et al., 2017). Accordingly, high-quality models depend on a common language and the mutual understanding of interdisciplinary topics. Establishing such a language is probably the first challenge but also a major contributor to the success of chromatography modeling endeavors.

## 4 Challenge II: Obtaining experimental data to set up the model

### 4.1 Properties of the chromatography system and column

#### 4.1.1 Porosity and pore size distribution

**FIGURE 1:**
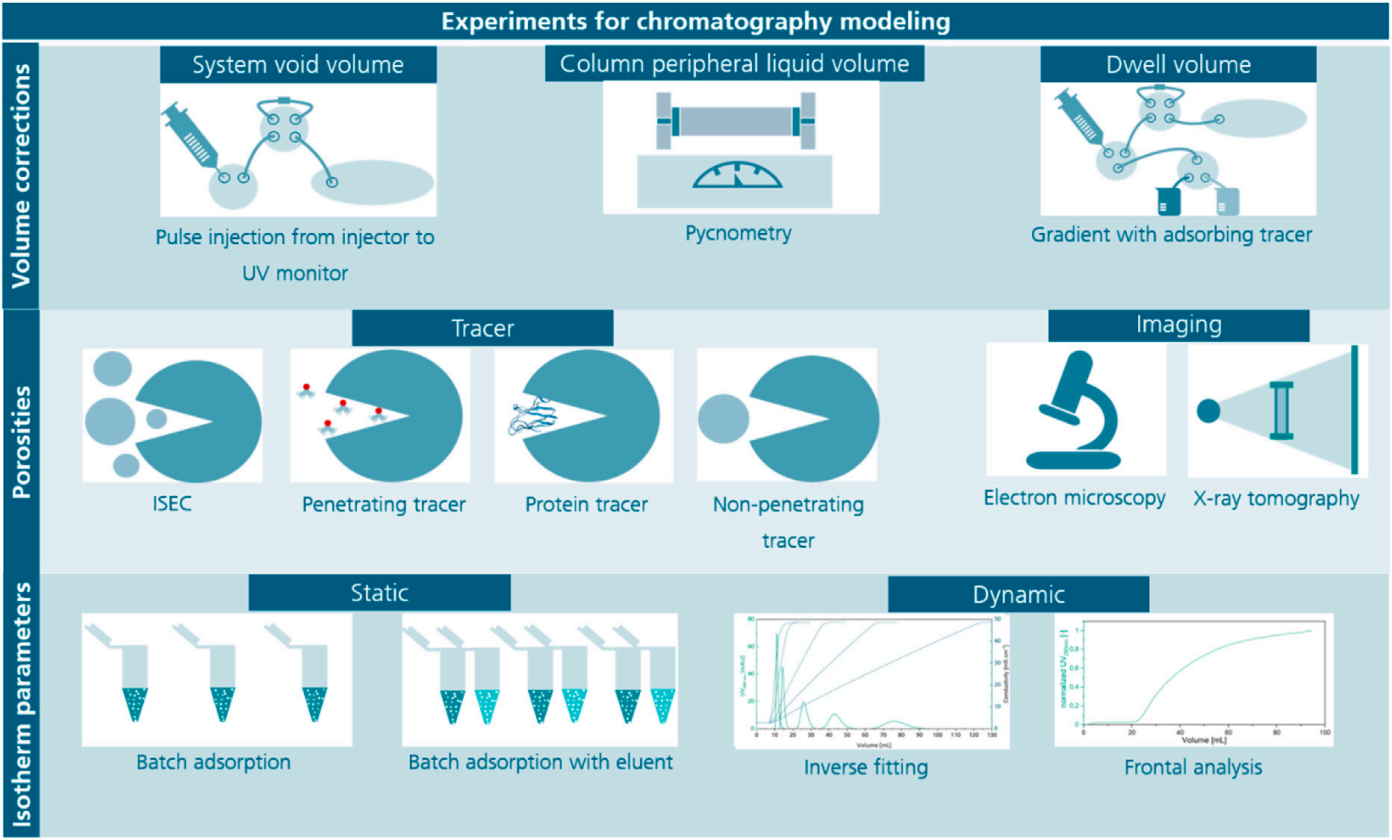
Overview of experiments for the determination of volume corrections of the chromatography system, porosities and isotherm parameters. Several volume corrections during chromatography modeling are necessary, here the most common methods are listed being pulse injections using an adsorbing tracer for the system void volume, pycnometry where the mass of the empty and water filled column is measured to determine the column peripheral liquid volume and the determination of the dwell volume using gradients with an adsorbing tracer while replacing the column with a zero-volume connector or a capillary restrictor. Methods for the determination of porosities are either a set of methods using different sized tracers, inverse size-exclusion chromatography (ISEC) or imaging methods such as electron microscopy or X-ray tomography, which require special equipment and complex evaluation. Tracers can be pore penetrating tracers or non-penetrating tracers either protein-based or synthetic. Lastly, methods for isotherm parameter determination mainly differ in static or dynamic approaches, which have individual advantages as suitability for screening (static) or high portion of information (dynamic).

A fundamental understanding of mass transport and binding equilibria is needed to establish models that can predict protein separation *in silico* (Miyabe and Guiochon 2003; Buyel et al., 2013; Vecchiarello et al., 2019; Kumar and Lenhoff 2020). Importantly, the corresponding parameters are interdependent during model calibration. For example, an inaccurately determined column porosity can distorted the value calculated for the equilibrium constant when fitting a chromatography model to a given peak (Heymann et al., 2022). Various methods have been proposed to determine equilibrium parameters (Shukla et al., 1998; Osberghaus et al., 2012a; Bernau et al., 2021) but the experimental determination of different types of liquid volumes in columns remains challenging, especially for packed-bed chromatography using porous, spherical beads as the stationary phase. These liquid volumes consist of inter-particle and intra-particle components (Figure 1) (Frank et al., 2022). When combined with the total (geometric) column volume and the particle solid volume, they can be used to calculate the inter-particle porosity (also known as column porosity) and the intra-particle porosity (also known as particle porosity). The latter values are needed to solve the partial differential equations of rate models that describe mass transport around the stationary phase (Wiesel et al., 2003; Orellana et al., 2009; Ghosh et al., 2014) and they can be combined with isotherms to model the binding of proteins (Brooks and Cramer 1992; Wang et al., 2016).

Whereas mass transfer around the particles is considered rapid because the mobile phase has a velocity of up to 7 m h-1 (Hahn et al., 2003a; Hahn et al., 2003b; Boi et al., 2020), diffusion into the resin pores is often assumed to be the rate-limiting step when a protein binds to the stationary phase (Schultze-Jena et al., 2019). Accordingly, resin particles with large pores, such as POROS with a pore diameter of up to 0.22 µm (Zhang et al., 2017), have been developed to increase pore diffusion while reducing steric hindrance (Anspach et al., 1989; Matlschweiger et al., 2019). Some manufacturers have also added porosity data to their documented resin specifications, which previously reported only the (dynamic) binding capacity, approximate particle diameter and average pore size. However, the information may not be available beforehand (i.e., for appropriate column selection) or may be limited to specific column types, such as *f(x) columns* (Cytiva, Sweden). This is why many researchers determine porosities experimentally, for example by combining small fully-penetrating tracers that can access all resin pores (such as acetone or salts) with non-penetrating tracers (such as dextran or spherical nanoparticles) that do not penetrate the resin pores at all (Halász and Martin 1978; DePhillips and Lenhoff 2000; Frank et al., 2022). Although such experiments can determine absolute porosities, they generally do not fully represent the pore fraction and thus the resin surface area available for the binding of macromolecules such as proteins, which partially penetrate the pores during separation (Table 2) (Pfister et al., 2015).

**TABLE 2:** Methods described in the literature for the determination of different column porosities.

Method	Porosity determination	Strength	Weakness	Reference
Inverse size exclusion chromatography with multiple, partially penetrating tracers	Pore size distribution	Determination of the pore size distribution and protein-specific porosity	Several tracers (proteins) required, tracer shape may distort results	DePhillips and Lenhoff (2000); Yao and Lenhoff (2004), 2006; Lubda et al. (2005); Forrer et al. (2008)
Injection of non-penetrating tracer	Inter-particle porosity	Simple method	Identifying suitable tracers can be challenging especially for resins with large pores (e.g., POROS)	Forrer et al. (2008); Osberghaus et al. (2012a); Wang et al. (2017b); Frank et al. (2022)
Injection of small penetrating tracers like salts or acetone	Total porosity	Simple method with readily available chemicals	Porosity values may not be representative for proteins	Osberghaus et al. (2012a); Huuk et al. (2016); Wang et al. (2017b)
Injection of protein penetrating tracer	Protein-specific porosity	Simple method, determines protein available pore space	Only reliable for proteins of similar size and shape, residual resin interactions possible	Zhang and Sun (2002); Forrer et al. (2008); Heymann et al. (2022)
Electron microscopy	Pore size, total porosity	Image of whole pore space, pore size distribution and porosity can be calculated	*Ex situ*, requires vacuum which potentially leads to deformation, expensive equipment	Hagel et al. (1996); Lubda et al. (2005); Yao et al. (2006)
X-ray computed tomography	Inter- and intra-particle porosity	Non-invasive and non-destructive imaging of packed beds	Special equipment necessary, intensive evaluation	Johnson et al. (2017); Johnson et al. (2018); Johnson et al. (2020)

Additional information is therefore required during modeling to make correct assumptions about parameters that are likely to be size-dependent and thus protein-specific, for example the ionic capacity (Λ) of the SMA model isotherm (Brooks and Cramer 1992). If not taken into account, the incorrect ionic capacity may be lumped into other parameters like the shielding factor σ (also known as the steric factor) during parameter fitting. This may be acceptable for certain chromatography settings, but could substantially distort model transfer during scale-up or switchover to another resin matrix. A pore size distribution would provide much more information in this context and has been determined for some resins (Yao and Lenhoff 2006).

Probing a packed column and resin with authentic proteins under non-binding conditions can provide information about the protein-specific accessible pore volume, which is essentially the same principle as size-exclusion chromatography (SEC) (Franke et al., 2010; Hong et al., 2012). One challenge during this type of analysis is the assignment of a size to the test proteins, due to (unexpected) oligomerization and/or non-spherical shapes, which might allow a protein to “squeeze” into pores smaller than their apparent hydrodynamic radius (Osberghaus et al., 2012b). The analysis of resins by electron microscopy can also reveal pore size distribution information (Zhu and Carta 2015), but the experimental conditions and results may not be comparable to those under authentic operational conditions due to the swelling, shrinking or deformation of the resin triggered by the media composition or compression during packing and operation (Conway and Sloane 1995; Nicoud 2015; Frank et al., 2022).

#### 4.1.2 Resin packing and wall effects

**FIGURE 2:**
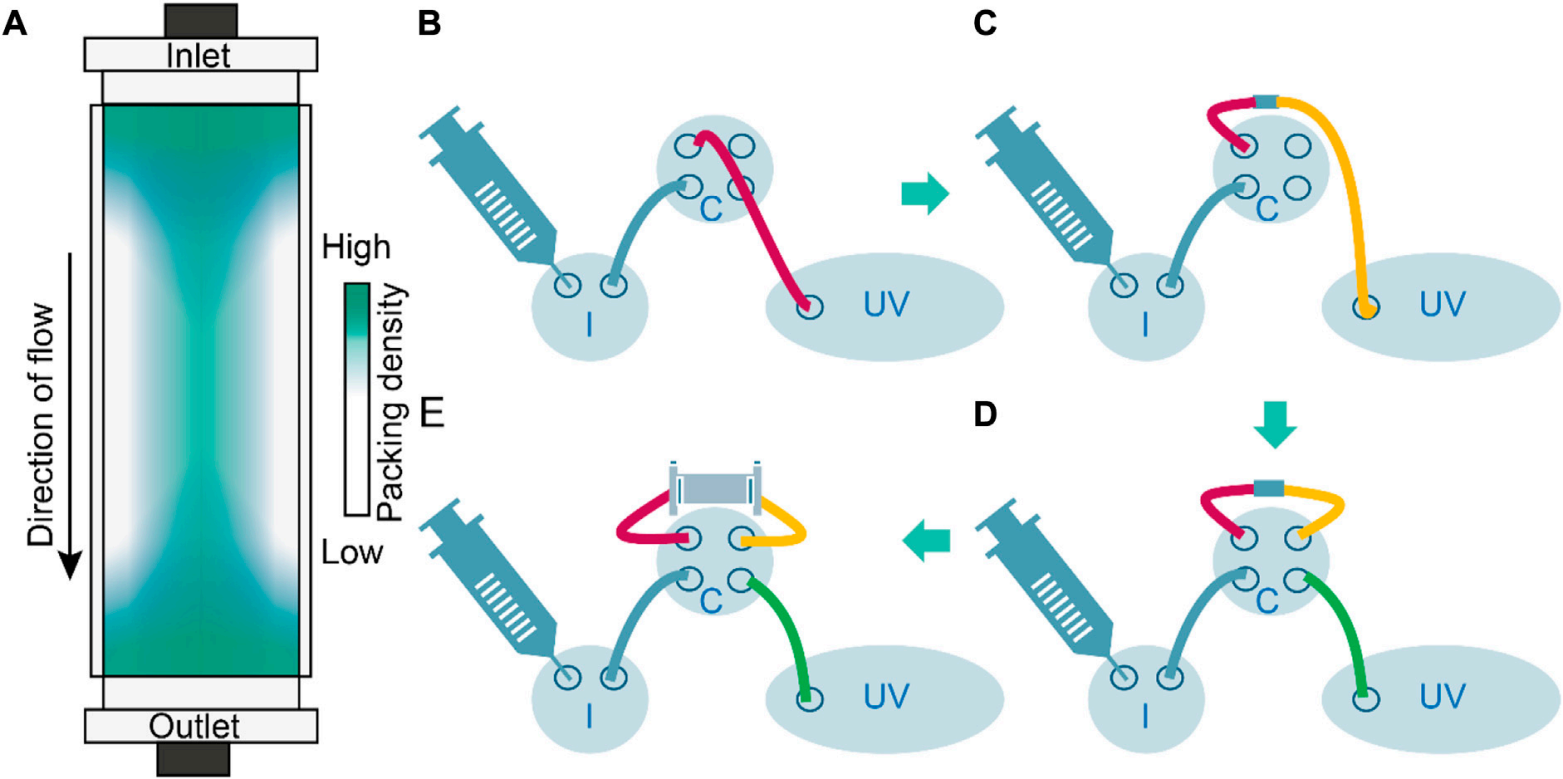
Column density distribution and analysis of pre and post-column void volumes to assess axial dispersion caused by instrument components. **(A)** Schematic representation of the resin density distribution in a packed-bed column. Packing is densest in the column center, especially at column inlet and outlet whereas wall effects reduce the density. **(B)** Determination of void volume and axial dispersion up to the column inlet by connecting injection valve I and column valve **(C)** through a column inlet tube (red) directly to the UV monitor (UV); V_pre-column_ = 21.7 × 10^−5^ L. **(C)** Determination of void volume and axial dispersion up to the column outlet by augmenting the setting in B with a column outlet tube (yellow) and a zero-volume connector; V_column_outlet_ = 22.8 × 10^−5^ L. **(D)** Determination of void volume and axial dispersion using a bypass setting (zero volume connector instead of a column) including a connection from column valve to UV monitor (green); V_zero_volume_connector_ = 30.0 × 10^−5^ L. **(E)** Determination of axial dispersion using a regular flow path including the column including the column peripheral liquid volume; V_column_outlet_ = 106.6 × 10^−5^ L.

The deformation of resin particles after column packing is indicated by the experimental inter-particle porosities of ∼12% (Frank et al., 2022), which is below the theoretical threshold of ∼26% for densely packed spheres (Conway and Sloane 1995). Such deformation can be expected because column packing typically involves linear flow rates of up to 7 m h^−1^ (Boi et al., 2020), which compress the resin particles beyond the point achieved by gravity settlement alone, typically by a factor of 1.15 for synthetic polymer resins (e.g., based on methacrylic polymers) that are considered to be semi-rigid (Baru 2003; Lee et al., 2015; Gebauer and Tschöp 2018). Although such packing stabilizes the chromatography bed (Dorn et al., 2017), it occurs predominantly at the inlet and outlet of the column (Dorn and Hekmat 2016; Dorn et al., 2017), thus distorting the flow regime in these regions. The analysis of fluid dynamics in packed beds can account for some degree of resin particle polydispersity (Shalliker et al., 2002; Püttmann et al., 2014) but the non-spherical shapes caused by dense packing are generally not considered (Bouhid de Aguiar et al., 2017). Importantly, ideal packing with uniform resin compression and the uniform arrangement of beads across the entire bed is impossible due to wall effects. These cause larger void volumes close to the column wall, where steric hindrance by the column corpus and friction increase the probability of random loose packing (Shalliker et al., 2000; Jin and Makse 2010; Bruns et al., 2012). In contrast, beads at the center of the columns can align freely and adopt a more ordered structure (Martinez et al., 2019). Accordingly, packing can be distorted in both the axial and radial directions (Figure 2). Such distortion is a topic of current research (Johnson et al., 2017; Dolamore et al., 2019; Johnson et al., 2020), and the results are included in some chromatography modeling environments such as CADET^7^. Anisotropic packing densities may limit the transferability of models calibrated on small-scale columns to process-scale equipment because the surface-to-volume ratio (and thus wall effects) will decrease with increasing scale (Püttmann et al., 2016). This scale effect is further aggravated by a decreasing column aspect ratio (height-to-diameter ratio) as the scale increases (Prentice et al., 2020).

#### 4.1.3 Volume corrections in chromatography modeling

Axial dispersion of solute peaks occurs in the packed bed of a column but also in column and system void volumes, also known as the system dead volume or column extra volume (Marek et al., 2018). This encompasses the liquid volumes contained in the tubing connecting the injector to the column inlet, and the column outlet to the UV detector, and liquid volumes within valves. A high system void volume increases the axial dispersion of an ideal rectangular plug injection outside the column, causing it to broaden into a Gaussian curve, ultimately reducing column efficiency (Iurashev et al., 2019), which is often defined through a (hypothetical) plate number *N*, a concept derived from distillation (Eq. 3):

$$N_{i}=5.54\;{\left(\frac{t_{R,\;i}}{w_{0.5,i}}\right)}^{2}$$ (3)

Where *N*_*i*_ is the number of plates in a column, t_R,i_ is the retention time of compound i and w_0.5,i_ is the corresponding peak width at half-height.

Based on a typical tubing diameter of 0.5 mm for fast protein liquid chromatography (FPLC) and a total tubing length of 321 mm, the system void volume contributed by the tubing is 6.3 × 10^−5^ L, which is minor compared to the system void volume contributed by injection and column valve (22.7 × 10^−5^ L ± 0.2 × 10^−5^, based on our measurements, n = 3) (Table 3). The dispersion caused by the system void volume is, however, negligible (<5%) for mass transfer parameters (e.g., molecular diffusion coefficient and effective particle diffusivity) (Gritti et al., 2006; Gritti and Guiochon 2014a). Still, this volume can become more relevant for efficient columns with >120,000 plates m^−1^, such as those used in ultra-high performance liquid chromatography (UHPLC), where it can reduce column efficiency 2.5-fold and ignoring it would substantially distort any chromatography model (Gritti and Guiochon 2014a). Accordingly, accounting for the individual sources of axial dispersion improves the scale-up and transferability properties of the models.

**TABLE 3:** Examples of system and column peripheral liquid volume as determined for an ÄKTA pure 25 L (nonstandard equipment: column valve kit V9-C and 5 mm UV flow cell) system equipped with an XK16/20 column (16 mm diameter, maximum bed height 200 mm).

Volume type	Contributing component	Volume [L]^a^
Pre-column void volume	Injection valves and column valve (only pre-column part)	18.6 × 10^−5^ ± 1.0 × 10^−5^
	Tubing (0.321 m)	3.1 × 10^−5^ ± < 0.1 × 10^−5^
Post column void volume	Column valve (only post-column part)	4.1 × 10^−5^ ± 0.8 × 10^−5^
	Tubing	3.2 × 10^−5^ ± < 0.1 × 10^−5^
	UV detector^b^	1.0 × 10^−5^ ± < 0.1 × 10^−5^
Column peripheral liquid volume^a^	Frits and connectors	76.6 × 10^−5^ ± 11.5 × 10^−5^
Total void volume	All	1.1 × 10^−3^ ± 0.1 × 10^−3^
Dwell volume^c^	Mixer and tubing	1.8 × 10^−3^ ± 0.1 ×10^−3^
	Command execution delay^d^	1.0 × 10^−5^

a volumes are given as ± standard deviation, n = 3 except for the column valve where n = 4.

b volume according to manufacturer’s information.

c volumes are given as average with ±standard deviation, for measurements with gradient lengths of 5, 10, 30 and 60 column volumes with n = 3 (N = 12).

d assuming a volumetric flow rate of 1 × 10^−3^ L min^−1^.

The system void volume is usually determined by replacing the column with a zero-volume connector and then injecting an acetone pulse (Schmidt-Traub et al., 2012; Marek et al., 2018; Desmet and Broeckhoven 2019; Bernau et al., 2021). The system void volume can then be calculated by multiplying the time difference between injection and detection at a UV monitor by the volumetric flow rate. By repeated executions of an according experimental method we found that the system void volume was 30.0 × 10^−5^ ± 0.2 × 10^−5^ L (n = 3). The corresponding coefficient of variation of 0.6% indicated a high reproducibility. The zero-volume connector method lumps dispersion by pre-column and post-column volumes into a single value (Vanderheyden et al., 2016; Desmet and Broeckhoven 2019). This can cause a systematic error of up to 60% for the axial dispersion (Gritti and Guiochon 2014b; Vanderheyden et al., 2016). Nevertheless, this effect is reduced if the system is operated in bind-and-elute mode due to the peak focusing effect of the packed bed (Prüß et al., 2003; Hong and McConville 2018) increasing the relevance of the dispersion by post-column volumes.

**FIGURE 3:**
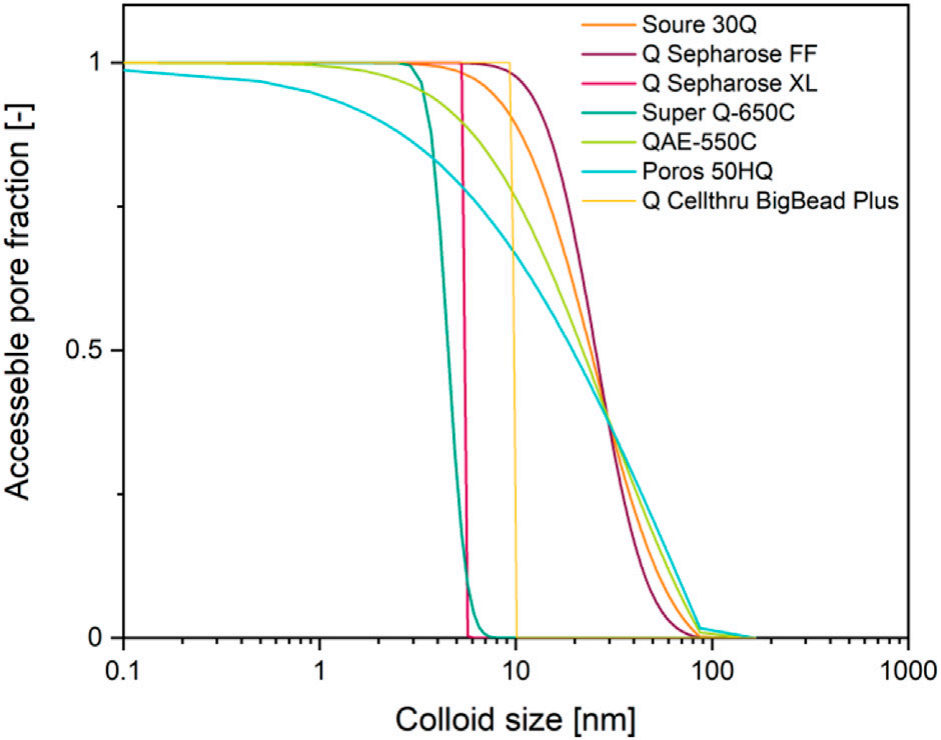
Accessible pore fraction as a function of colloid size and depending on the pore size distribution of common anion exchange resins. Pore size distribution data (Yao and Lenhoff 2006) were used to build a cumulative sum and then normalized using the highest value.

Alternatively, the pre-column and post-column volumes can be determined separately by stepwise addition of instrument components and respective void volume measurements (Figure 3) (Gilar et al., 2017; Desmet and Broeckhoven 2019). Other approaches determine the column-based dispersion first, then subtracting it from the overall dispersion to calculate the system-related dispersion (Desmet and Broeckhoven 2019).

The column dispersion can be determined by injecting a fluorescent analyte onto a column and then quantifying the band broadening between column inlet and outlet (Evans and McGuffin 1988). The system dispersion can be obtained by measuring the total dispersion as a function of column bed height (i.e. for columns of different length) and extrapolating this function to a bed height of zero (i.e. the *y*-intercept) (Roper and Lightfoot 1995; Guo and Frey 2010). However, these methods require special equipment such as a pre-column fluorescence detector or several columns and are often labor intensive (Evans and McGuffin 1988; Desmet and Broeckhoven 2019).

An additional volume that is important but often overlooked during chromatography modeling is the column peripheral liquid volume (Table 3), which includes the liquid volume in the frits and connectors at the column inlet and outlet (Gritti et al., 2015). This volume can be measured by pycnometry, which in this case is weighing an empty column equipped with frits but devoid of resin and weighing the same column filled with water (Jiang et al., 2014). The column peripheral liquid volume then corresponds to the mass difference multiplied by the density of water at the experiment temperature, minus the packed-bed volume (Marek et al., 2018). This method is straightforward in principle but can be challenging in practice if the bed height needed to calculate the packed-bed volume is difficult to determine. For example, an uncertainty of 0.5 mm in bed height measurement for a column 16 mm in diameter with a bed height of 155 mm (3.1 × 10^−2^ L bed volume) causes a volume difference of 2 × 10^−4^ L, which corresponds to ∼26.2% of the actual column peripheral liquid volume. The bed height may be well known for pre-packed columns but it may be difficult to obtain empty reference columns for comparison, i.e., empty but assembled columns in pre-packed format are often not available by manufacturers. Ultimately, the influence of the column peripheral liquid volume depends on the ratio of the column peripheral liquid volume to the bed volume. For an XK 16/20 column, i.e., a column with 16 mm diameter and a maximum bed height of 200 mm, this ratio can vary from 0.767 for a bed volume of 1 ml to 0.024 for the maximum bed volume of 3.1 × 10^−2^ L.

Finally, the initiation of an elution gradient will involve some delay, also known as the dwell volume, between the execution of the command in the system control software and the formation of the elution gradient in the column (Guillarme et al., 2008). The processing time in the software is negligible [<50 ms (Aichernig et al., 2019)], so the delay predominantly results from the physical distance between the mixer outlet and the column inlet as well as the diameter of the tubing used to bridge this distance (Table 3). The dwell volume is often measured using two solutions, one with and one without a UV-adsorbing tracer. The latter is used at first to equilibrate the chromatography system with all columns detached. Then, the tracer-containing solution is injected to form a gradient of desired length (Bos et al., 2021). The time delay between gradient initiation and the increase in UV signal is multiplied by the volumetric flow rate to derive the dwell volume. This volume is then used to correct the time shift between the programmed and actual gradient onset in order to obtain retention volumes or times that are comparable in terms of the solvent composition across different system settings (Silver 2019).

### 4.2 Protein-specific isotherm parameters

The use of pure proteins for bind-and-elute experiments, which are needed to determine the protein-specific parameters of binding kinetics like the equilibrium constant between the protein and stationary phase, can improve the reliability of parameter determination (Borrmann et al., 2011; Wang et al., 2017a; Moreno-González et al., 2021). Such experiments can be conducted using static or dynamic methods (Figure 1). In static methods, protein dilution series of known concentrations are exposed to defined quantities of resin, for example in a 96-well plate format, for a period of time considered sufficient for protein binding to reach a steady-state equilibrium (Ghose et al., 2007; Moreno-González et al., 2021), often 24–48 h. The protein concentrations in the supernatant are then determined, and any differences compared to the starting concentrations are used to determine equilibrium constants of (static) binding (Guiochon 2002; Seidel-Morgenstern 2004). The calculation can be improved by closing the protein mass balance, for example by washing the resin, then adding an eluent and measuring the protein concentration in this liquid fraction (Seidel-Morgenstern 2004). Whereas the general workflow is simple, the implementation can be prone to errors because it is difficult to aliquot resins in a reproducible manner and to account for residual liquids that unintentionally cause dilution, for example due to the sedimentation of resin particles (Coffman et al., 2008). Such static methods often lack precision, but the main drawback is that, by design, the equilibrium constant is determined for static conditions that are not representative of the dynamic binding that occurs during process-scale chromatography, reflecting the continuous flux of the mobile phase through a packed-bed, monolithic or membrane-based column (Nachman et al., 1992; Ostryanina et al., 2002; Ghose et al., 2007; Carta 2012; Faraji et al., 2015). When comparing literature data (Table 4), binding capacities obtained from batch adsorption are ∼1.5-fold higher compared to the same constants determined under dynamic conditions. Therefore, batch adsorption is often used for initial screening experiments but not to calibrate the chromatography models (Khosravanipour Mostafazadeh et al., 2011; Moreno-González et al., 2021).

**TABLE 4:** Static and dynamic binding capacities obtained for different target proteins and chromatography resins.

Resin [-]	Manufacturer [-]	SBC [mg mL^−1^]	DBC [mg mL^−1^]	Target protein	Information [-]	Reference [-]
UNOsphere SuPrA	Bio-Rad	39	24.5	IgG lambda-like polypeptide 1	P15814	Perez-Almodovar and Carta (2009)
UNOsphere S	Bio-Rad	93	61.2	mAb	∼150 kDa, pI ∼ 8.9, deaminated	Tao et al. (2011a)
Capto S	Cytiva	207	122.2	mAb	∼150 kDa, pI ∼ 8.9, deaminated	Tao et al. (2011b)
SP Sepharose FF	Cytiva	79	25	IgG human	∼150 kDa	Yoshimoto et al. (2016)
SP Sepharose HP	Cytiva	72	68	IgG human	∼150 kDa	Yoshimoto et al. (2016)
SP Sepharose FF	Cytiva	83.7	88.3	Lysozyme	P00698	Yoshimoto et al. (2016)
SP Sepharose HP	Cytiva	110.2	70	Lysozyme	P00698	Yoshimoto et al. (2016)
Proprietary resin “j” (coupled Fractogel EMD)	Cytiva	31	37	IgG human (Beriglobin)	IgG_1_ (61%), IgG_2_ (28%), IgG_3_ (5%), IgG_4_ (6%) and IgA (1%)	Hofer et al. (2011)
Proprietary resin “k” (coupled Fractogel EMD)	Cytiva	34	32	IgG human (Beriglobin)	IgG_1_ (61%), IgG_2_ (28%), IgG_3_ (5%)	Hofer et al. (2011)
					IgG_4_ (6%) and IgA (1%)	
Proprietary resin “l” (coupled Fractogel EMD)	Cytiva	57	68	IgG human (Beriglobin)	IgG_1_ (61%), IgG_2_ (28%), IgG_3_ (5%)	Hofer et al. (2011)
					IgG_4_ (6%) and IgA (1%)	
Q Sepharose FF	Cytiva	108	3	α-lactalbumin	P00711	Yang et al. (2002)
Q Sepharose FF	Cytiva	115	110	Thyroglobulin	F1RRV3	Yang et al. (2002)
Q Membran	Merck	2.97 ± 0.37	4.3 ± 0.03	α-lactalbumin	P00711	Yang et al. (2002)
Q Membran	Merck	9.8 ± 0.75	11 ± 0.9	Thyroglobulin	F1RRV3	Yang et al. (2002)
Modified regenerated cellulose membrane	Proprietary	15.63	5.3 ± 0.5	Bovine serum albumin	P02769	Liu et al. (2017)
Sartobind S	Sartorius Stedim	70.0	17.8	Bovine serum albumin	P02769	Tatárová et al. (2013)
	Biotech					
Sartobind D	Sartorius Stedim	62.9	52.1	Bovine serum albumin	P02769	Tatárová et al. (2013)
	Biotech					
Sartobind Q	Sartorius Stedim	49.0	41.0	Bovine serum albumin	P02769	Tatárová et al. (2013)
	Biotech					
Sartobind S	Sartorius Stedim	70.0	30.4	Lysozyme	P00698	Tatárová et al. (2013)
	Biotech					
Sartobind S	Sartorius Stedim	84.8	34.5	Myoglobin	P68082	Tatárová et al. (2013)
	Biotech					
Sartobind D	Sartorius Stedim	51.0	48.8	human serum albu-min	P02768	Tatárová et al. (2013)
	Biotech					
Sartobind Q	Sartorius Stedim	43.1	40.1	human serum albu-min	P02768	Tatárová et al. (2013)
	Biotech					

SBC, static binding capacity; DBC, dynamic binding capacity; IgG, immunoglobulin; mAb, monoclonal antibody; FF, fast flow; HP, high performance.

Dynamic methods require fewer experiments than static methods, but rely on a continuous flux applied over the (packed-bed) column and the protein concentration at the column outlet must be measured over time, for example during elution. Expensive equipment is also necessary, typically an FPLC system (e.g., ÄKTA series devices) and corresponding analytics such as in-line UV and conductivity determination (Seidel-Morgenstern 2004; Luca et al., 2020; Kumar et al., 2021). One widely-used approach is the inverse method (Osberghaus et al., 2012a; Hahn et al., 2016a; Hahn et al., 2016b), which fits isotherm parameters to an experimental elution peak by minimizing the discrepancies between experimental and simulated peaks (Dose et al., 1991; Leweke and Lieres 2018). Typically, two chromatograms are necessary to fit, for example, the equilibrium constant *k*_*eq*_ and the characteristic charge *ν* of the SMA isotherm (Osberghaus et al., 2012a; Bernau et al., 2021). The amount of protein required for such an experimental series is ∼0.6 mg, depending on the quality of the UV monitor. Using competitive isotherms for multicomponent elution as well as fluorescent proteins or tags alleviates the need for highly pure protein during model calibration (Seidel-Morgenstern 2004; Baumann et al., 2015). In our hands, purities of >50% are typically required to obtain a Gaussian curve-shaped peak for fitting (Bernau et al., 2021), yet higher purities may be necessary depending on the type, number and individual abundance of non-target protein impurities. The presence of such impurities may result in shoulders or even several peak maxima, which in the simplest case can falsify the fitted transport parameters, and in the worst case can result in incorrect protein-specific isotherm parameters due to the selection of inappropriate peak properties, e.g., maxima, for fitting (Pirrung et al., 2018). Nevertheless, the inverse method can determine parameter values for multicomponent isotherms, even for separation factors of 0.9–1.1 (Kaczmarski 2007; Hahn et al., 2016a), if the corresponding proteins do not interact with each other.

Elution experiments for inverse fitting can be complemented with frontal experiments, where a column is loaded until protein breakthrough, to increase the precision of parameter values or to determine additional model parameters such as the shielding factor of the SMA isotherm (Osberghaus et al., 2012a). The latter requires no limitation on liquid film and pore diffusion mass transfer, which would otherwise distort the chromatogram shape and thus the isotherm parameter values (Persson et al., 2004; Seidel-Morgenstern 2004; German 2012). If frontal analysis alone is used for isotherm parameter determination, multiple breakthrough experiments are needed spanning a range of feed protein concentrations (e.g., 1–100 mM) to determine corresponding stationary phase concentrations at equilibrium (Andrzejewska et al., 2009; Kamarei et al., 2014). Each stationary phase concentration represents a data point for the fitting of isotherm parameters. However, frontal experiments often require 50–200 mg of pure protein per run (Yao and Lenhoff 2006; Hardin et al., 2009; Lin et al., 2016) when using common 1-ml columns, and this amount can be difficult to generate, especially during early process development. Data analysis is also more complex for dynamic methods. Typical office computation power can be sufficient for the parameter fitting task, but the specific approach can have a substantial impact on the precision and speed of the parameter fitting (Section 5.2.1) (Saleh et al., 2020; Jäpel and Buyel 2022).

## 5 Challenge III: Effects not yet represented in chromatography models

Until recently, chromatography modeling largely focused on the development of formulae that adequately describe mass transfer and protein sorption, such as mechanistic descriptions of isotherms, pore diffusion and film diffusion (Gotmar et al., 1999; Miyabe and Guiochon 2003; Kumar and Lenhoff 2020; Schmidt-Traub et al., 2020). As chromatography models evolve, attention is increasingly shifting from these broad topics to finer details such as resin batch-to-batch variability, scalability, and the transferability of models to resins with the same ligand but a different particle size, pore size (distribution), linker chemistry or base matrix (Bernau et al., 2021). These aspects influence model robustness, thus affecting regulatory compliance (Saleh et al., 2021c) and transferability, because the experiments required to set up such models are laborious (Saleh et al., 2020). For example, reducing the particle size by a factor of 0.6 will typically increase the resolution (Eq. 4) and separation factor by 1.4 (Eq. 5) (Biba et al., 2014), whereas inter-particle porosity is likely to decrease due to an increase in packing density reflecting the lower particle diameter (Sohn and Moreland 1968).

$$R_{s}=2\left(\frac{t_{R_{1}}+t_{R_{2}}\;}{w_{b_{1}}+w_{b_{2}}}\right)$$ (4)

$$\alpha =\frac{t_{R_{2}}}{t_{R_{1}}}$$ (5)

Where R_s_ is the resolution of the solutes achieved with the chromatography setting, t_R1_ and t_R2_ are the retention times of solutes 1 and 2 respectively, with solute 1 eluting first, w_b1_ and w_b2_ being the corresponding peak widths at baseline, and α is the separation factor.

However, correlations between particle size and changes in resolution can be predicted, for example by calibration using multiple columns packed with same resin of the same ligand and matrix type but different particle sizes. It can be difficult to account for changes in pore size (distribution) *a priori* because mass transfer into and within resin pores is a limiting factor, which in turn is highly specific to the molecules that are separated (Persson et al., 2004; Wang et al., 2017b). The effects of resin matrix chemistry, such as shortening the spacer arm of Sepharose FF to increase protein retention (DePhillips et al., 2004), should be addressed in future experiments because all chromatography model isotherms that we are aware of are limited to protein–ligand interactions, thus assuming resin matrix inertia (Brooks and Cramer 1992; Nfor et al., 2010; Wang et al., 2016; Schmidt-Traub et al., 2020). Accordingly, any matrix effect would be lumped into the isotherm parameters, limiting the transferability of the model as discussed above.

In this context, chromatography models also do not account for the indirect binding of proteins. For example, some host cell proteins (HCPs) bind to recombinant protein products such as monoclonal antibodies, and then co-purify during chromatography, not because the HCPs bind to the ligand, but due to their interaction with the recombinant protein, a phenomenon known as ‘hitchhiking’ (Baik et al., 2019). Unexpected mass transfer limitations due to the restricted pore diffusion may reflect the presence of a hitchhiking protein because the protein–protein interaction will increase the apparent size of the target protein. The modeling of hitchhiking phenomena would be useful in the context of biopharmaceutical process development because the abundance of hitchhiking HCPs in a purified recombinant protein can form part of the quality product profile, representing a critical quality attribute (Baik et al., 2019; Mouellef et al., 2021). Accounting for hitchhiking would require that protein–protein interactions are routinely included in chromatography models. This can be achieved to some extent using docking software as long as information is available about the structure of the HCP during separation (Busetta et al., 1983; Kitchen et al., 2004; Ciemny et al., 2018; Bitencourt-Ferreira et al., 2019), but this requires long computation times of up to 11 h (Moal et al., 2017; Porter et al., 2019). Although protein structures are increasingly accessible, for example *a priori* predictions generated by AlphaFold (Jumper et al., 2021), the entire workflow can be prohibitively time-consuming and expensive given that several hundred HCPs may be present in the feed in addition to the target protein, especially if the host cells have to be disrupted (Buyel et al., 2013; Joucla et al., 2013; Park et al., 2017).

**FIGURE 4:**
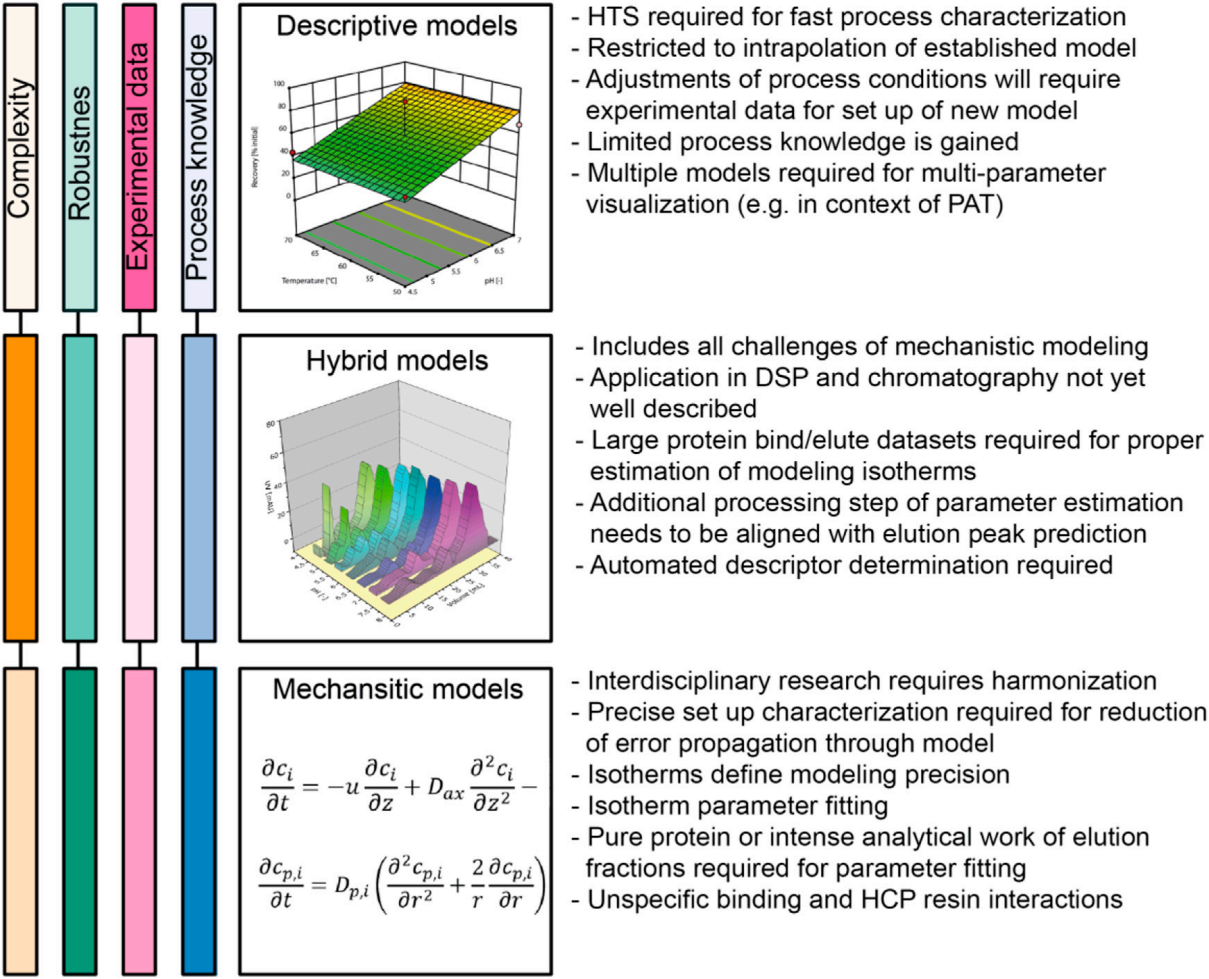
Comparison of data-driven, mechanistic and hybrid chromatography models. Color intensities correlate with the peculiarity/gravitas of the respective criterion for the respective model type. Here, complexity includes the mathematical frame of the model as well as efforts for model implementation, whereas robustness is defined by the accuracy of model predictions and the capability of extrapolation outside the calibration scale. Experimental data accounts for the number of experimental effort required for model calibration and validation and process knowledge summarizes the mechanistic information and process understanding provided by the model. DSP, Downstream processing; HCP, host cell protein; HTS, high-throughput screening; PAT, process analytical tools.

Another effect that is rarely accommodated in modeling chromatography is a limited pore accessibility due to steric hindrance based on the size and shape of a protein or particle, for example a virus-like particle (Coquebert de Neuville et al., 2013; Pfister et al., 2015). If such colloids have a hydrodynamic diameter of ∼100 nm (>10,000 kDa) (Johnson 2000; Goicochea et al., 2011), almost all pores of widely-used resins such as Q Sepharose FF are inaccessible to them (Figure 4) (Hagel et al., 1996; DePhillips and Lenhoff 2000; Yao and Lenhoff 2006). As a result, the dynamic binding capacity can be unexpectedly low, for example 3.8 × 10^−4^ mg L^−1^ in case of Hepatitis B virus surface antigen virus-like particles binding to DEAE Sepharose FF (Yu et al., 2014). Similarly, mass transfer into, within and out of the pores can be slow for large colloids, causing extensive tailing during elution (Schmidt-Traub et al., 2012).

## 6 Challenge IV: Data driven modeling–Feature extraction and model quality assessment

Data-driven approaches, like QSAR modeling, are regression tasks associated with typical regression challenges. On one hand, regression relies on experimental data for calibration, which can be a limiting factor (e.g., proteins for which isotherm parameters have been determined). On the other hand, the number of features (descriptors) capturing protein properties can be much larger than the number of data points. This phenomenon is described as the ‘curse of dimensionality’ and leads to challenges such as data sparsity, multicollinearity, and overfitting (Altman and Krzywinski 2018). Feature reduction is therefore a typical first step in model building, and is based on feature selection or feature extraction. Feature extraction methods create new features by transforming the original feature space into a lower-dimensional space (Khalid et al., 2014), for example using the popular PCA approach, which is a simple non-parametric linear transformation. Feature extraction is especially useful if a feature set does not contain properties detectable by a given learning algorithm, because this method aggregates information from all features without risking the erroneous removal of relevant features, which can occur during feature selection. The downside of feature extraction is that the new features can be difficult to interpret, and the contribution of the original attributes is often lost (Janecek et al., 2008).

Feature selection chooses a suitable subset of features and is classified into 1) filter methods, which calculate a score for each feature separately and choose the features with the best score; 2) wrapper methods, which fit a supervised learning model onto feature subsets and choose the subset with the best performance; and 3) embedded methods, where the feature selection algorithm is integrated as part of the learning algorithm, e.g., by penalizing the usage of to many features (Bommert et al., 2020). Feature selection and feature extraction are widely used to reduce dimensionality and therefore reduce the risk of regression tasks to overfit, e.g., during QSAR modeling, ultimately improving model generalizability (Alsenan et al., 2020).

Experimental variability causes noise in some features (i.e., attributes, e.g., peak retention time) and target values (i.e., the regressand, e.g., SMA parameters) reducing the identifiability of relevant features by feature selection and correlation during feature extraction. Also, for the regression task, the predictive performance of a regression model depends on the model complexity (e.g., linear vs. non-linear), the amount of noise, and possible noise filtering methods. For example, Martin et al. compared 5 different regression algorithms on 20 real-world data sets and injected noise levels from 5% to 30% and found that the RMSE deteriorated by 67%–100% (Martin et al., 2021). Given that especially QSAR models are evaluated using a test dataset extracted from the same noisy primary dataset, QSAR model evaluation is error-prone and model performance may be underrated (Gupta and Gupta 2019; Kolmar and Grulke 2021). As a result, the predictive quality of QSAR models typically decreases with increasing noise (Janecek et al., 2008; Gupta and Gupta 2019).

Similarly, the choice of regression method can affect the model quality. Whereas MLR is a classical approach, easy to implement and interpret (Eriksson et al., 2003), other regression methods have been used in recent QSAR applications, including decision trees, ANNs, SVMs, random forests, and *k*-nearest neighbors algorithms, as well as deep learning approaches such as convolutional neural networks (Lin et al., 2020). For example, 77 regression approaches representing 19 families of methods were recently assessed on 83 data sets. The results showed that for small and difficult datasets (i.e., < 5000 entries, linear regression model R^2^ < 0.6) the penalized linear regression achieved the best results [Friedman-Rank of R^2^ score: 8.45, the score is explained elsewhere (López-Vázquez and Hochsztain 2019)] followed by a random forest (Friedman-Rank of R^2^ score: 15.3) (Fernández-Delgado et al., 2019). Sparse data, i.e. only one or a few data points within in each area of the feature space, pose another challenge for prediction tasks because for any new data point there are most likely no similar data points in the training set. One way to compensate for small datasets, and thus sparse data, is semi-supervised learning, where accessory unlabeled data (i.e., data points with known features but unknown target values) can improve predictions (van Engelen and Hoos 2020). Specifically, these unlabeled data are often available in large quantities and are incorporated into supervised models or the models are directly trained using both labeled and unlabeled data (Frumosu and Kulahci 2018). The idea of semi-supervised models is to use labeled data points in a supervised manner to update the model while unlabeled data points are used to minimize the difference in the predictions between similar training examples.

Regardless of the model building approach, the predictive power must be assessed. For one, cross-validation is a widely-used internal validation method that iteratively develops models on different data subsets (training datasets) while the remaining data points (test dataset) are used for validation. Leave-one-out cross-validation is the simplest and most popular approach because it makes best use of the underlying data, which is especially important in small datasets (Wu et al., 2010). However, leave-many-out cross-validation gives a more reliable estimate of model predictive accuracy by omitting ∼30% of the data points in small datasets (20–30 data points) and even more of the data points in larger datasets (Gramatica 2007). If enough reliable data are available, the best way to confirm model accuracy is to test the performance externally on a sufficiently large number of data points that have not been used for model building and internal validation (Gramatica 2007).

Finally, *y*-randomization or *y*-scrambling can be used to compare a regression model with other models using randomized target values, which allows the detection of overfitting (Kaneko 2019). For example, if a regression model has predictive properties similar to a *y*-scrambled counterpart, there is a strong likelihood of overfitting in the original model. The challenge with all evaluation methods is to define thresholds that would indicate a ‘good’ model, because the quality metrics are continuous and transitions between ‘good’ and ‘bad’ are therefore fluid (Schober et al., 2018). One option is to select different thresholds for external and internal validation metrics. For example, determination coefficient thresholds can be set to R^2^ > 0.7 for the training data set whereas a leave-one-out threshold of Q_LOO_^2^ > 0.6 is defined for the test data set in order to obtain a high quality QSAR model (Chirico and Gramatica 2012).

## 7 Challenge V: Mechanistic model fitting–Parameter value optimization in multi-dimensional spaces

Parameters for mechanistic models can be determined using three different approaches: 1) correlation-based approaches using batch equilibrium data; 2) correlation-based approaches using chromatography data [applicable for specific isotherms only, e.g., SMA (Rüdt et al., 2015)]; and 3) inverse fitting using modeling software.

The first option, which is applicable only to isotherm parameters and not transport parameters, fits isotherm equations to measured batch adsorption data (Xu and Lenhoff 2008, 2009; Nfor et al., 2010). However, the setting is typically assessed at equilibrium state of adsorption and desorption, which can take more than a day to establish for some systems (Kumar and Lenhoff 2020) and is longer than the contact time for most column-based operations. Therefore, the transferability of batch adsorption results to dynamic chromatography conditions using a continuous mobile phase flux is often limited.

The second option estimates parameters based on the features of experimental chromatograms and theoretically derived equations without the use of modelling software. This includes the determination of porosities based on the retention volume of tracer experiments (Carta and Jungbauer 2010) as well as methods for the determination of isotherm binding parameters based on the peak positions during linear gradient elution (Yamamoto et al., 1983; Brooks and Cramer 1992; Shukla et al., 1998; Yamamoto and Miyagawa 1999; Rüdt et al., 2015; Lee et al., 2017).

The third option involves the use of modelling software to inversely fit parameters by incrementally adjusting model parameters within a mechanistic model simulation until the simulated chromatogram best matches the experimental target data (Osberghaus et al., 2012a; Heymann et al., 2022). This method is the most computationally expensive of the three, but it allows the creation of complete chromatogram predictions using the fitted parameter values and resulting model. It is also applicable to all chromatography conditions that can be modeled even if no correlation-based method is available (Saleh et al., 2020), including multicomponent competitive binding in the non-linear range of the SMA isotherm. Inverse fitting can become challenging however if the number of parameter values to be fitted simultaneously increases and may outnumber the features in the input data (e.g., peak shape properties of gradient elution chromatograms) or if inter-dependencies between parameters exist because the underlying numerical problem becomes ill-conditioned, as was encountered for pH-dependent SMA (Saleh et al., 2020), HIC (Wang et al., 2016) and MMC (Nfor et al., 2010) isotherms. This can be improved if individual parameters can be identified *a priori* using correlation approaches based on chromatography data, e.g., by applying the Yamamoto method that enabled the reduction of the number of parameters from 32 to 7 as described before (Saleh et al., 2020). A combination of approach 2) to estimate parameters in the linear range of the isotherm and the inverse fitting 3) to estimate the non-linear parameters has been proven as robust method for model calibration (Saleh et al., 2020). Parameter estimation algorithms can also get stuck in local minima and take impractically long timescales to converge, e.g., several days (Heymann et al., 2022). This can be overcome by using modern genetic algorithms (Heymann et al., 2022) or Bayesian inference (Briskot et al., 2019; Jäpel and Buyel 2022). The sum of squared differences between the simulated chromatogram and the experimental chromatogram is frequently used as an objective function to assess the quality of the model prediction. This objective function is sensitive to the relative timings of the elution peaks rather than their shapes, leading to recommendations for a new objective function system specifically designed for chromatography modeling (Heymann et al., 2022). This system utilizes the “shape” objective function, which is measured by the maximum of the Pearson correlation coefficient between the simulated and the experimental chromatogram over a continuous range of time offsets. The system also includes the difference in timing and peak height as objective functions. All three objective functions can be evaluated using chromatograms and/or over their first derivatives. The approach is more robust against experimental measurement errors such as dispersion in external hold-up volumes or systematic pump-delays compared to methods based on sum of squared differences (Heymann et al., 2022).

## 8 Conclusion

Chromatography modeling can be achieved by the application of descriptive, mechanistic or hybrid approaches, each with distinct advantages and limitations (Figure 4). The choice of modeling approach depends on the quantity and quality of available data, mechanistic understanding of the separation task and the type of expertise and resources accessible to a development project team. Purely data-driven descriptive models provide a fast-forward description of the process, but the resulting knowledge is difficult to extend beyond the design space or to other purification processes. A deep understanding of separation fundamentals is required for mechanistic models preventing an easy access but providing substantial gains once mastered. Hybrid models therefore seem to emerge as an attractive middle ground, replacing mechanistic aspects with data-driven counterparts where acceptable in terms of predictive quality while keeping the reliability of mechanistic models where necessary. We have identified several major challenges that should be considered when setting up chromatography models, and recommend further scientific evaluation to overcome the hurdles that currently restrict the broad application of modeling in industry settings and especially in academia.

The number of chromatography resins and modes of chromatography available commercially or established in academia is steadily increasing (Rühl et al., 2018; Tustian et al., 2018; Knödler et al., 2019), which will translate into an increasing number of isotherms to be deployed and modeling frameworks should be ready to implement such new isotherms quickly. The development of new isotherms for resin modalities not used as frequently as IEX, for example MMC and uncommon affinity resins, will be an interesting research area and application field for hybrid models. In this context, non-specific binding, including protein-matrix interactions but also protein-protein-matrix interactions will open up a whole new aspect of chromatography modeling.

Ultimately, combining individual chromatography models into models of multi-column chromatography processes will require an adaptation of the modeling approaches to handle the increased complexity, higher variability in experimental outcomes and therefore the additional experimental effort needed to generate suitable datasets for model validation (Behere and Yoon 2020; Guo et al., 2020). Similarly, the computational effort will increase for the modeling of more than one column due to a combinatorial explosion (Behere and Yoon 2020; Guo et al., 2020). All the more important will it be to establish standardized methodologies for model quality assessment that could form the basis for a good modeling practice (GMoP) complementing other GxP guidelines in the context of biopharmaceutical production (Rischawy et al., 2019; Roush et al., 2020; Saleh et al., 2021b; Rajamanickam et al., 2021). We think that such a standardization process will greatly profit from open access chromatography databases for sharing experimental data in a uniform and annotated manner suitable for model building and testing. Such a constantly growing dataset may ultimately facilitate precise characterizations for PAT applications or QbD implementation once predictive errors are sufficiently low.

## 9 Future perspectives

Modeling chromatography is a highly interdisciplinary approach that has attracted great interest, especially when applied in biopharmaceutical DSP, which requires a profound understanding of the process and adaptability based on the precise prediction of altered process conditions (Saleh et al., 2021c). Within the last decade, interest in such models has increased, mainly due to the QbD initiative but also due to the economic advantages for companies in the pharmaceutical sector (Shekhawat et al., 2016). The application of data-driven, mechanistic and hybrid models requires adequate software-based solutions e.g., for the implementation of the mass transport and adsorption models in order to characterize particle movement and interaction with ligands. This can be achieved using software tools like CADET (Leweke and Lieres 2018) or Cytiva’s GoSilico Chromatography Modeling Software, which will surely become more versatile and accessible in the future. The body of literature presenting valuable mechanistic modeling reports for late-stage DSP steps is increasing (Wang et al., 2016; Saleh et al., 2020; Saleh et al. 2020; Saleh et al. 2021b; Saleh et al. 2021c; Kumar et al., 2021) and so is the market for customer-friendly modeling solutions. The applicability of chromatography models may further increase in the future, for example through hybrid models that use a mechanistic framework for mass transport and sorption but implement descriptive models for the respective calibration. Ultimately, chromatography modeling has the potential to accelerate bioprocess development and reduce the associated costs.

## Footnotes

1
www.cytivalifesciences.com/en/us/news-center/cytiva-acquires-gosilico-to-strengthen-digital-capabilities-in-bioprocessing-10001.

2
https://cadet.github.io/master/modelling/unit_operations/general_rate_model.html.

3
https://cadet.github.io/master/modelling/unit_operations/2d_general_rate_model.html.

4
https://cadet.github.io/master/modelling/binding/index.html.

5
https://www.ypsofacto.com/services-chemical-software-chromworks.

6
https://github.com/modsim/CADET/issues/14.

7
https://www.sartorius.com/download/549152/poster-multi-column-chromatography-process-modelling-for-process- performance-prediction-data.pdf.

## Abbreviations

DoE, design of experiments; DSP, downstream processing; EMA, European Medicines Agency; FDA, US Food and Drug Administration; HCP, host cell proteins; HIC, hydrophobic interaction chromatography; MCC, multi-column chromatography; MMC, multi-modal chromatography; MPC, model-predictive control; PAT, process analytical technology; QbD, quality by design; QSAR, quantitative structure–activity relationship; RMSE, root-mean-square error; SDM, scale down model; USP, upstream processing; UV, ultraviolet.
